# Effects of Direct Renin Blockade on Renal & Systemic Hemodynamics and on RAAS Activity, in Weight Excess and Hypertension: A Randomized Clinical Trial

**DOI:** 10.1371/journal.pone.0169258

**Published:** 2017-01-24

**Authors:** A. J. Kwakernaak, L. C. Roksnoer, H. J. Lambers Heerspink, I. van den Berg-Garrelds, G. A. Lochorn, J. H. van Embden Andres, M. A. Klijn, H. Kobori, A. H. J. Danser, G. D. Laverman, G. J. Navis

**Affiliations:** 1 Department of Medicine, Division of Nephrology, University of Groningen, University Medical Center Groningen, The Netherlands; 2 Department of Medicine, Division of Vascular Medicine and Pharmacology, Erasmus Medical Center, Rotterdam, The Netherlands; 3 Department of Clinical Pharmacology, University of Groningen, University Medical Center Groningen, The Netherlands; 4 General Practitioner Practice Gorecht, Hoogezand, The Netherlands; 5 General Practitioner Practice Boterdiep, Groningen, The Netherlands; 6 Department of Pharmacology, Kagawa University, School of Medicine, Miki, Kita District, Kagawa, Japan; 7 Department of Internal Medicine, Division of Nephrology, ZGT Hospital Almelo, Netherlands; Osake University Graduate School Of Medicine, JAPAN

## Abstract

**Aim:**

The combination of weight excess and hypertension significantly contributes to cardiovascular risk and progressive kidney damage. An unfavorable renal hemodynamic profile is thought to contribute to this increased risk and may be ameliorated by direct renin inhibition (DRI). The aim of this trial was to assess the effect of DRI on renal and systemic hemodynamics and on RAAS activity, in men with weight excess and hypertension.

**Methods:**

A randomized, double-blind, cross-over clinical trial to determine the effect of DRI (aliskiren 300 mg/day), with angiotensin converting enzyme inhibition (ACEi; ramipril 10 mg/day) as a positive control, on renal and systemic hemodynamics, and on RAAS activity (n = 15).

**Results:**

Mean (SEM) Glomerular filtration rate (101 (5) mL/min/1.73m^2^) remained unaffected by DRI or ACEi. Effective renal plasma flow (ERPF; 301 (14) mL/min/1.73m^2^) was increased in response to DRI (320 (14) mL/min/1.73m^2^, P = 0.012) and ACEi (317 (15) mL/min/1.73m^2^, P = 0.045). Filtration fraction (FF; 34 (0.8)%) was reduced by DRI only (32 (0.7)%, P = 0.044). Mean arterial pressure (109 (2) mmHg) was reduced by DRI (101 (2) mmHg, P = 0.008) and ACEi (103 (3) mmHg, P = 0.037). RAAS activity was reduced by DRI and ACEi. Albuminuria (20 [9–42] mg/d) was reduced by DRI only (12 [5–28] mg/d, P = 0.030).

**Conclusions:**

In men with weight excess and hypertension, DRI and ACEi improved renal and systemic hemodynamics. Both DRI and ACEi reduced RAAS activity. Thus, DRI provides effective treatment in weight excess and hypertension.

**Trial Registration:**

Dutch trial register, registration number: 2532 www.trialregister.nl

## Introduction

The prevalence of weight excess has been steadily rising over the past decades and shows no sign of abating yet, thereby becoming a major global health problem of the 21st Century [1,2]. The association between weight excess and hypertension is widely recognized, and linked to an increased risk for long-term cardiovascular and renal damage [3–7]. The increased renal risk associated with weight excess and hypertension is only partly explained by the elevated blood pressure as such, and additional factors such as insulin resistance and an unfavorable renal hemodynamic profile have been implicated [8–11].

Weight excess is associated with distinct renal hemodynamic abnormalities, that are prominent in subjects with overt obesity, but already apparent in the overweight range, with an elevated filtration fraction (FF) as a common denominator [12]. The latter may reflect glomerular hypertension that contributes to long-term renal damage, as shown in animal experiments [13]. We previously reported on the consistent association between higher body mass index (BMI) and higher FF, and moreover, showed that higher FF is independently associated with worse long-term outcome in renal transplant recipients, supporting a role of higher FF as a renal risk factor in humans [14].

Blockade of the renin-angiotensin-aldosterone system (RAAS) reduces blood pressure and exerts specific renal hemodynamics effects, with a reduction in FF, and provides long-term renoprotection in patients with renal disease [15,16]. Accordingly, the renal hemodynamic actions of RAAS blockade may be of benefit especially in subjects with weight excess and hypertension. In line, ACEi exerts beneficial effects on renal hemodynamics in overweight and obesity [17]. There is data to suggest that DRI might be particularly effective in modulating renal RAAS [18]. However, the effect of DRI on renal hemodynamics and RAAS activity has not been tested so far in subjects with weight excess and hypertension. We therefore assessed the effect of DRI in maximal dose, with maximal dose ACEi as a positive control, on renal hemodynamics, twenty-four hour ambulant blood pressure, and on RAAS activity parameters in men with weight excess and hypertension.

## Material and Methods

### General trial information

This randomized, double-blind, cross-over clinical trial was performed between January 2011 and June 2012 at the Department of Medicine, Division of Nephrology, of the University Medical Center Groningen (UMCG), Groningen, The Netherlands (Trial protocol in S1 Text). Primary outcome measure of the trial were renal hemodynamics (glomerular filtration rate: GFR, effective renal plasma flow: ERPF, and filtration fraction: FF) and systemic blood pressure (systolic blood pressure: SBP, diastolic blood pressure: DBP, and mean arterial pressure: MAP) as measured by twenty-four hour ambulatory blood pressure measurement (ABPM). Secondary outcome measures of the trial were RAAS activity (renin concentration and activity, aldosterone concentration, aldosterone/renin concentration ratio, and angiotensinogen concentration) and volume status (extracellular fluid volume: ECV). The trial was conducted according to the ethical principles of the Declaration of Helsinki and Good Clinical Practice (GCP), and was approved by the Independent Medical Ethics Committee of our University Medical Center (METc-number: 2010/228). The trial is registered at the Dutch trial register (www.trialregister.nl; trial registration number: 2532). All participants provided written informed consent.

### Trial participants

We screened consecutive Caucasian men with weight excess and essential hypertension from our outpatient clinic for nephrology and hypertension, and from two local general practitioner clinics. Inclusion criteria were a BMI between >27 and ≤35 Kg/m^2^, essential hypertension (WHO criteria; either treated with antihypertensive medication or untreated ambulant systolic and/or diastolic blood pressure ≥140 and/or ≥90 mmHg, respectively [19]), normal renal function (endogenous creatinine clearance ≥90 mL/min/1.73m^2^), and normo- or microalbuminuria (urinary albuminuria excretion <300 mg/day). For safety reasons we excluded subjects with off-treatment systolic and diastolic blood pressure of ≥180 and ≥110 mmHg, respectively, and subjects with a history of cardiovascular disease (myocardial infarction, angina pectoris, percutanous transluminal coronary angioplasty, coronary artery bypass grafting, stroke, heart failure (stage I-IV of the New York Heart Association classification). Other main exclusion criteria were: diabetes mellitus, active malignancy, any medication and/or surgical or medical condition that might alter absorption, distribution, metabolism, or excretion of medication, history of hypersensitivity or contraindication to trial medication or radio-labeled tracers, history of angioedema, autonomic dysfunction, participation in any other clinical investigation within three months prior to start of the trial, blood or plasma donation within 3 months prior to initial dosing, and history of either drugs or alcohol abuse.

### Trial protocol

Subjects that were treated with antihypertensive medication prior to start of the trial were first enrolled in a 6-week wash-out period in which prior antihypertensive medication was stopped. NSAIDs were not allowed and discontinued at start of the wash-out period (n = 2). Other non-trial drugs were kept stable during the trial. Consecutively, subjects were randomly assigned (1:1) to either a 6-week treatment period with angiotensin converting enzyme-inhibition (ACEi; ramipril 10 mg/day) and aliskiren-placebo or direct renin-blockade (DRI; aliskiren 300 mg/day) and ramipril-placebo, according to the double-dummy principle, in a cross-over fashion. An independent pharmacist randomized treatment sequences using a computer. The randomization code remained secret during the entire study and all patients, investigators, and health care providers were blinded, except for the pharmacist. Dose of DRI and ACEi were chosen on basis of their maximal recommended dose according to European Medicines Agency (www.ema.europa.eu). After completion of the first treatment period, subjects enrolled in an 8-week wash-out period after which the second treatment period started.

Subjects visited our outpatient clinic for nephrology and hypertension at baseline, and after completion of the wash-out period and the 2 treatment periods for clinical assessment (body weight, and adverse events), measurement of renal hemodynamics (GFR, ERPF, and FF), monitoring of 24-hour ambulant and office blood pressure (SBP, DBP, and MAP), volume status (ECV), and for blood and 24-hour urine sampling for measurement of RAAS parameters (renin concentration and activity, aldosterone concentration, aldosterone/renin concentration ratio, and angiotensinogen concentration), and routine hematology and biochemistry variables. Subjects were instructed to take trial medication once daily, in the morning, except when renal hemodynamic measurements were performed. Furthermore, subjects were instructed to adhere to a regular protein and sodium diet (being 1.1 g/Kg body weight/day and 200 mmol/day, respectively). No structured follow-up of subjects after trial completion was performed.

### Trial measurements and calculations

#### Renal hemodynamics

Constant infusion of radio-labeled tracers, ^125^I-iothalamate, and ^131^I-hippurate, was used to measure GFR and ERPF, respectively, with subjects being in a quiet room, in a semi-supine position. After drawing a blank blood sample, a priming solution containing 0.04 mL/Kg body weight of the infusion solution (0.04 MBq of ^125^I-iothalamate and 0.03 MBq of ^131^I-hippurate) plus an extra bolus of 0.06 MBq of ^125^I-iothalamate was given at 08:00 hours, followed by infusion at a rate of 12 mL/hour. In order to attain stable plasma concentrations of both tracers, a 2-hour stabilization period followed, after which baseline measurement started at 10:00 hours. The clearances were calculated as (U×V)/P and (I×V)/P, respectively. U×V represents the urinary excretion of the tracer, I×V represents the infusion rate of the tracer, and P represents the tracer value in plasma at the end of each clearance period. This method corrects for incomplete bladder emptying and dead space, by multiplying urinary clearance of ^125^I-iothalamate with the ratio of the plasma and urinary clearance of ^131^I-hippurate [20,21]. FF was calculated by dividing GFR by ERPF, and expressed as percentage. Renal vascular resistance (RVR) was calculated as the ratio of MAP (calculated with blood pressures measured during renal hemodynamic measurements as described further on), and renal blood flow, the latter being ERPF multiplied by 1 minus hematocrit. ECV was calculated using the distribution volume of ^125^I-iothalamate, as described previously [22].

To comply with common practice in literature, we indexed renal hemodynamic parameters, except FF, for body surface area (BSA). However, as this can induce bias when analyzing renal hemodynamics in overweight and obese subjects, we additionally repeated analyses for crude (mL/min) values of GFR, ERPF and RVR.

#### Systemic hemodynamics

Twenty-four hour ambulant blood pressure measurements (Spacelabs Medical^®^, Inc. Issaquah, WA, USA) were performed one day prior to the measurement of renal hemodynamics. At baseline, blood pressure was measured at both arms to check for presence of a clinical significant difference in blood pressure (present in none of the subjects). We measured upper-arm circumference at baseline to custom-fit cuff size, and subjects were instructed to place their arm in a resting position during blood pressure measurement. Blood pressure cuffs were applied by either a trained technician or by A.J.K. Blood pressure was measured every 30 minutes during both day- and night time. A measurement was noted as unsuccessful when number of recordings was less than 80% (1 patient at end of the DRI treatment period).

In addition, blood pressure was measured after completion of the 2-hour stabilization period during renal hemodynamic measurement, at 1-minute intervals by an semi-automatic device (Dinamap^®^, G.E. Medical Systems, Milwaukee, WI, USA), with subjects being in a quiet room, in a semi-supine position, and in a fasting condition. We used the mean of the single last 4 readings (last reading was omitted as subjects may react to the nurses entering the room). We expressed blood pressure as systolic, diastolic and mean arterial pressure, the last being calculated as diastolic pressure plus one third of pulse pressure.

#### RAAS parameters

Fasting blood samples were obtained at start of renal hemodynamic measurement, after a minimum semi-supine rest of 15 minutes. Twenty-four hour urine was collected at the day prior to the hospital visit. Blood samples for measurement of RAAS activity parameters were immediately put on ice, centrifuged at 3000 RPM for 10 min at 4°C, and subsequently frozen on liquid nitrogen and stored at −80°C until analysis. Plasma renin activity was measured by determining angiotensin I generation at 37°C in the presence of angiotensinase inhibitors. Detection limit of this assay was 0.03 pmol angiotensin I /mL/hr, and the coefficient of variance (CV) was 11%. Plasma and urinary renin concentration were measured with an immunoradiometric assay (Renin III; Cisbio, Gif-sur-Yvette, France), with a detection limit of 1 pg/mL, and a coefficient of variance (CV) of 7%. Plasma and urinary aldosterone were measured with a radioimmunoassay (Coat-a-Count, Diagnostics Product Corporation, Siemens, LA, CA, USA). This assay has a detection limit of 11 pg/mL, and a CV of 8%. Plasma angiotensinogen was measured as the maximum quantity of angiotensin I that was generated during incubation with excess recombinant renin. The detection limit of this assay was 0.50 pmol/mL, and the CV was 10%. We expressed plasma angiotensinogen as pg/mL (multiplying by its molecular weight of 65 kDa). Urinary angiotensinogen was measured with a commercial angiotensinogen ELISA (IBL International, Hamburg, Germany), with a detection limit of 0.01 ng/mL, and a CV of 5%. Urinary measurements of RAAS parameters were performed in 24-hour urine samples and expressed as excretion rates per 24-hour. Aldosterone/renin concentration ratio was calculated by dividing aldosterone concentration by renin concentration. Urine/plasma concentration ratios of renin, aldosterone and angiotensinogen were calculated by dividing urinary concentration by plasma concentration, and were multiplied by 100%.

#### Other measurements and calculations

Routine hematology and biochemistry variables were measured within 2 hours after blood and urine sampling. Proteinuria and albuminuria were measured with a turbidimetric assay using benzethonium chloride (Modular, Roche Diagnostics, Mannheim, Germany). Values of urinary protein concentration were below detection limit (0.1 g/L) in 4, 3 and 4 subjects at baseline, DRI, and ACEi, respectively, and was set at 0.05 g/L in order to calculate urinary excretion rate. Urinary albuminuria concentration was below detection limit (1 mg/L) in one subject during ACEi, and was set at 0.5 mg/L. Blood electrolytes, lipids, glucose, and urinary electrolytes were measured using an automated multianalyser (Modular, Roche Diagnostics, Mannheim, Germany). Creatinine clearance was calculated from creatinine concentration in plasma and 24-hour urine sample. Body mass index (BMI), as a measure of overall obesity, was calculated by dividing body weight by height squared (kg/m^2^). Obesity was defined as BMI > 30 kg/m^2^. Body surface area (BSA) was calculated according to the DuBois-DuBois formula [23]. Waist and hip circumference were measured on bare skin, at the natural indentation between the 10^th^ rib and iliac crest and at the region of the trochanter major, respectively. Waist circumference was measured after an overnight fast and at end of normal expiration to avoid influence of stomach content and respiration phase on measurements. Waist-to-hip ratio (WHR) was calculated as waist circumference divided by hip circumference.

### Statistical analysis

We expected subjects to present with a mean unindexed ERPF of 444 mL/min at baseline. As ample size calculation (two-sided T-test) was performed on basis of a hypothesized increase in ERPF of 47 mL/min with DRI and 29 mL/min with ACEi [15,24], with a standard deviation in ERPF response of 17 mL/min. In order to give the trial 90% power to detect a statistically significant increase in ERPF during both ACEi and DRI (α = 0.05) we calculated that a total of fourteen subjects had to complete the cross-over design sequence. We aimed to randomize 16 subjects at start of the trial to anticipate on a dropout rate of 10%.

Analyses were performed after database was locked. Paired T-tests were used to determine treatment response. Non-normally distributed variables were log_10_-transformed before analysis. In addition, we analysed data by linear mixed model analysis, including a Bonferroni correction, with renal and systemic hemodynamics and RAAS-parameters as dependent variables, subjects as a random factor, and treatment (ACEi or DRI) and sequence (start with either ACEi or DRI) as well as their interaction (treatment x sequence) as fixed factors, to account for repeated measurements and tot check for presence of any potential carry-over effects of treatment. As this analysis did not essentially change results we only present paied T-tests. Renal hemodynamic and blood pressure measurements were essential similar for baseline and wash-out period, and therefore only baseline data are shown. Data are given as mean with standard error of mean (SEM) when normally distributed, and otherwise as geometric mean with 95% confidence interval (95% CI). Data was analyzed using SPSS version 20.0 (SPSS Inc., Chicago, IL) and GraphPad Prism version 5 (GraphPad Software Inc., San Diego, CA). Statistical significance was assumed at the 5% level of probability.

## Results

### Trial population

**Fig 1:**
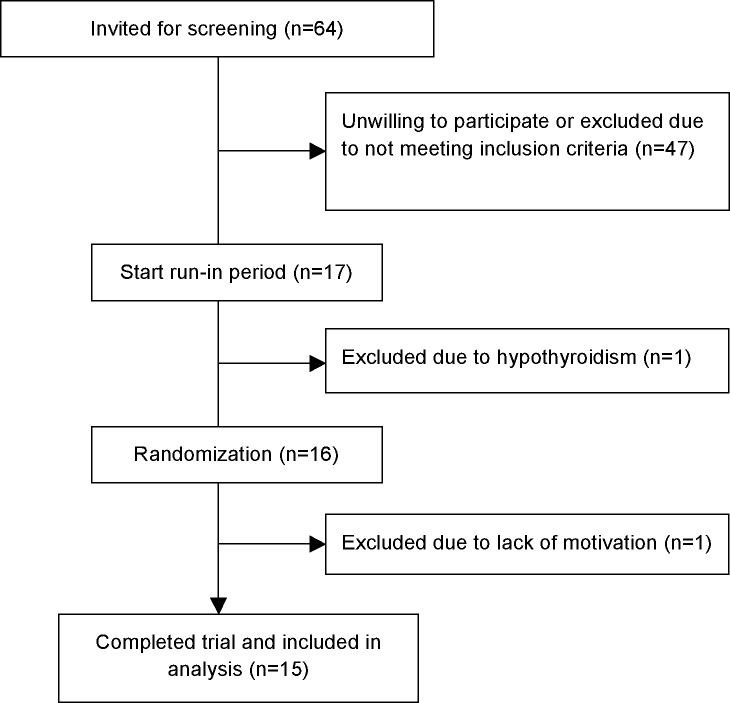
Flow chart of inclusion.

We invited 64 overweight/obese hypertensive male subjects for an information visit at the outpatient clinic of which 17 subjects responded and were subsequently found eligible for participation. During the run-in period, 1 patient was excluded because of asymptomatic subclinical hypothyroidism with high anti-thyroid peroxidase auto-antibodies for which thyroid hormone substitution was indicated. The remaining 16 subjects were randomized. After baseline measurement, 1 subject refused further participation due to lack of motivation and was therefore excluded. A total of 15 subjects completed the trial and were included in analyses (Fig 1).

Subject characteristics are shown in Table 1. By default, subjects were hypertensive and overtly overweight, with 47% being obese (defined as BMI > 30 Kg/m^2^). Fasting plasma glucose, HbA1C, and cholesterol levels were all within normal limits. Before trial enrollment, 10 (67%) subjects used 1 [0–2] antihypertensive medication, being either ACEi (n = 7), ARB (n = 3), diuretics (n = 7), or calcium channel blockers (n = 1). No statistical differences in subject characteristics were noted when stratified for treatment of initiation (S1 Table). Compliance to ACEi and DRI capsules during both trial periods, as assessed by pill counts, was > 85% in all but one subject that had a compliance of 76% during the ACEi treatment.

**Table 1:** Subject characteristics (n = 15).

Age (years)	58 (3)
Male gender, n (%)	15 (100%)
BMI (Kg/m^2^)	30 (1)
Obesity, n (%)	7 (47%)
Office SBP (mmHg)	149 (5)
Office DBP (mmHg)	93 (3)
AHM prior to inclusion, n [range]	1 [0–2]
Waist circumference (cm)	108 (2)
Hip circumference (cm)	103 (2)
WHR	1.06 (0.02)
HbA1C (%)	5.8 (0.16)
Fasting plasma glucose (mmol/L)	5.9 (0.3)
Total cholesterol (mmol/L)	4.9 (0.2)
LDL cholesterol (mmol/L)	3.2 (0.2)
HDL cholesterol (mmol/L)	1.2 (0.1)

Data are shown as mean (SEM) or as geometric mean (95% CI) when indicated. Office blood pressure was measured with semi automatic blood pressure device (Dinamap^®^).

Abbreviations: BMI: body mass index; SBP: systolic blood pressure; DBP: diastolic blood pressure; AHM: antihypertensive medication; WHR: waist-to-hip ratio; LDL: low density lipoprotein; HDL: high density lipoprotein.

### Renal hemodynamics

**Fig 2:**
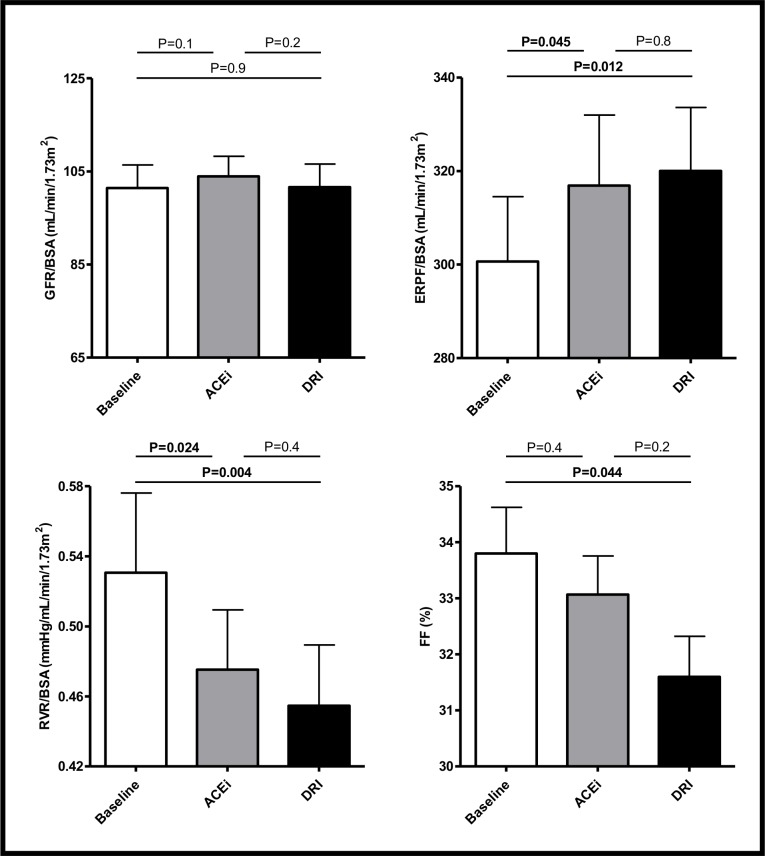
Renal hemodynamic parameters at baseline and after 6-week treatment with ACEi and DRI. Renal hemodynamic data indexed for BSA. Data shown as mean (SEM). Abbreviations: BSA: body surface area; GFR: glomerular filtration rate; ERPF: effective renal plasma flow; FF: filtration fraction.

BSA-indexed renal hemodynamic parameters at baseline and after six week treatment with DRI and ACEi are shown in Fig 2. Mean (SEM) GFR/BSA at baseline was 101 (5) mL/min/1.73m^2^ and remained essentially unaffected by DRI (102 (5) mL/min/1.73m^2^, P = 0.9) and by ACEi (104 (4) mL/min/1.73m^2^, P = 0.1). ERPF/BSA was significantly increased in response to DRI (320 (14) mL/min/1.73m^2^, P = 0.012) and ACEi (317 (15) mL/min/1.73m^2^, P = 0.045) compared to baseline (301 (14) mL/min/1.73m^2^). Both DRI (0.45 (0.03), P = 0.004) and ACEi (0.47 (0.03), P = 0.024) reduced RVR/BSA compared to baseline (0.53 (0.05)), although FF was only significant reduced in response to DRI treatment (DRI: 32 (0.7)%, P = 0.044 and ACEi: 33 (0.7)%, P = 0.4, respectively) compared to baseline (34 (0.8)%). Essentially similar results were found when we repeated analyses with crude GFR, ERPF and RVR (Table 2; see S1 Table for data stratified by treatment of initiation). Although there was a tendency of stronger effects on renal hemodynamics with DRI, the difference in response of ERPF, RVR and FF between DRI and ACEi was not significant. Furthermore, linear mixed model analysis did not essentially change results.

**Table 2:** Renal hemodynamics and systemic blood pressure measurements at baseline and after treatment with ACEi and DRI.

	Baseline	ACEi	DRI
**Crude renal hemodynamic data**			
GFR (mL/min)	130 (7)	133 (6)	131 (7)
ERPF (mL/min)	386 (19)	406 (20)*	411 (18)**
RVR (mmHg/mL/min)	0.67 (0.05)	0.60 (0.04)*	0.58 (0.04)**
FF (%)	34 (0.8)	33 (0.7)	32 (0.07)*
**Semi-automatic blood pressure data (DINAMAP**^®^**)**			
SBP (mmHg)	149 (5)	142 (4)**	136 (3)**
DBP (mmHg)	93 (3)	87 (3)	85 (3)*
MAP (mmHg)	112 (4)	106 (3)*	102 (3)**

Data are shown as mean (SEM). Abbreviations: BSA: body surface area; GFR: glomerular filtration rate; ERPF: effective renal plasma flow; FF: filtration fraction, RVR: renal vascular resistance; SBP: systolic blood pressure; DBP: diastolic blood pressure; MAP: mean arterial pressure.

* P<0.05 vs. baseline

** P<0.01 vs. baseline.

### Systemic hemodynamics

**Fig 3:**
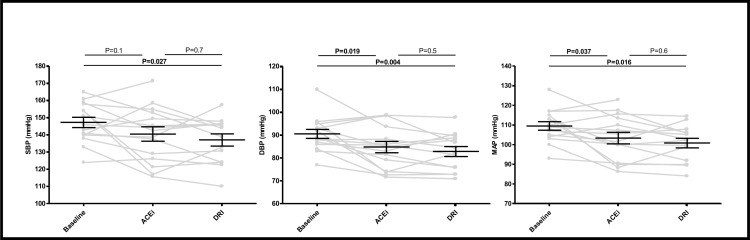
Blood pressure measured by ABPM at baseline and after 6-week treatment with ACEi and DRI. Individual data are shown as well as mean (SEM). ABPM measurement was unsuccessful in one patient during DRI treatment due to insufficient number of recordings (<80%). Abbreviations: SBP: systolic blood pressure; DBP: diastolic blood pressure; MAP: mean arterial pressure.

Fig 3 shows data on twenty-four hour ambulant blood pressure measurements at baseline and after six week treatment with DRI and ACEi. Mean (SEM) baseline systolic blood pressure (147 (3) mmHg) was significantly reduced in response to DRI (137 (4) mmHg, P = 0.027) and nominally reduced in response to ACEi (140 (4) mmHg, P = 0.1). Baseline diastolic blood pressure (91 (2) mmHg) was significantly reduced by both DRI (83 (2) mmHg, P = 0.004) and ACEi (85 (2) mmHg, P = 0.019). Consequently, both DRI (101 (2) mmHg, P = 0.008) and ACEi (103 (3) mmHg, P = 0.037) reduced MAP compared to baseline (109 (2) mmHg). Results obtained by twenty-four hour ambulant blood pressure measurements were confirmed by office blood pressure measurement at time of renal hemodynamic measurements using a semi automatic device (Table 2). There was no significant difference in blood pressures response between DRI and ACEi. Essential similar results were found with linear mixed model analysis.

### RAAS activity

**Fig 4:**
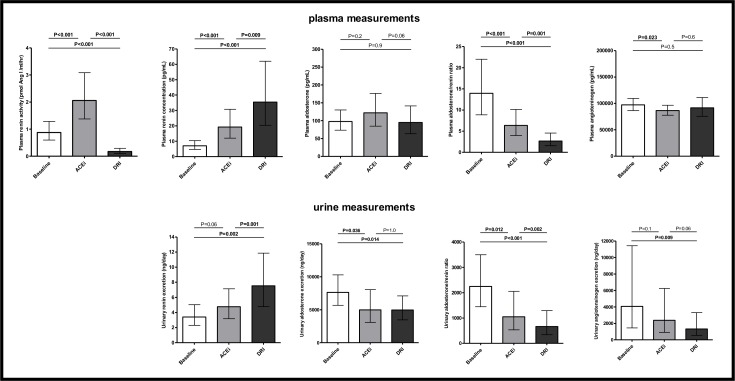
RAAS parameters in plasma (upper panel) and urine (lower panel) at baseline and after 6-week treatment with ACEi and DRI. Data shown as geometric mean (95% CI).

**Fig 5:**
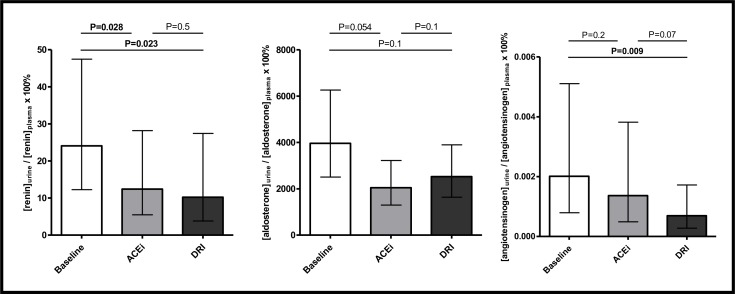
Renal angiotensinogen handling at baseline and after 6-week treatment with ACEi and DRI. Data shown as geometric mean (95% CI).

Fig 4 shows data on RAAS parameters at baseline and after six week treatment with DRI and ACEi. All subjects had a pronounced rise and reduction in plasma renin concentration and aldosterone/renin ratio, respectively, confirming good compliance to trial medication. Geometric mean (95% CI) of plasma renin activity at baseline (0.9 [0.6–1.3] pmol Ang I /mL/hr) was significantly reduced in response to DRI (0.2 [0.1–0.3] pmol Ang I /mL/hr, P<0.001), and significantly increased in response to ACEi (2.1 [1.4–3.1] pmol Ang I /mL/hr, P<0.001). Plasma renin concentration and urinary renin excretion at baseline (7.0 [4.7–10.4] pg/mL and 3.4 [2.3–5.0 ng/day], resp.) were significantly increased by DRI (35.4 [20.3–61.9] pg/mL and 7.5 [4.8–11.9] ng/day, resp.; both P<0.001 vs. baseline) and to a lesser extent by ACEi (19.2 [12.0–30.7] pg/mL and 4.8 [3.2–7.1] ng/day, resp.; both P<0.001 vs. baseline). Urinary excretion of aldosterone at baseline (5672 [5672–10290] ng/day) was significantly reduced by DRI (4969 [3475–7106] ng/day, P = 0.014) and ACEi (4987 [3084–8062] ng/day, P = 0.036), without affecting plasma aldosterone levels (98 [73–130] pg/mL, 95 [64–142] pg/mL, and 122 [84–176] pg/mL for baseline, DRI and ACEi, resp.; P>0.05 for both DRI and ACEi). Consequently, aldosterone/renin concentration ratio in both plasma and urine at baseline (14.0 [8.9–22.0] and 2248 [1444–3499], resp.) were significantly reduced by DRI (2.7 [1.6–4.5], P<0.001) and 660 [339–1288], P = 0.012) and to a lesser extent by ACEi (6.4 [4.0–10.2], P<0.001 and 1046 [533–2054], resp. P<0.001). Plasma angiotensinogen at baseline (97295 [86438–109515] ng/mL) was not affected by DRI (91688 [75576–111235] ng/mL, P = 0.6), but significantly reduced by ACEi (86488 [77523–96490] ng/mL, P = 0.023). In contrast, urinary angiotensinogen at baseline (4067 [1448–11425] ng/day) was significantly reduced by DRI (1325 [531–3304] ng/day, P = 0.009) and not by ACEi (2378 [907–6237] ng/day, P = 0.1). Data on urine/plasma concentration ratios of renin, aldosterone and angiotensinogen are shown in Fig 5. The urine/plasma concentration ratio of renin and angiotensinogen at baseline (24 [12–47]% and 0.0020 [0.0008–0.0051]%, resp.) were significantly reduced in response to DRI (10 [4–27]%, P = 0.023 and 0.0007 [0.0003–0.0017]%, P = 0.009, resp.). The urine/plasma concentration ratio of aldosterone was neither affected by DRI nor by ACEi. DRI had overall a stronger effect on RAAS activity parameters, which reached statistical significance for plasma and urinary renin concentration (P = 0.009 and P = 0.001 compared to ACEi, resp.) and plasma and urinary aldosterone/renin concentration ratio (P = 0.001 and P = 0.002 compared to ACEi, resp.).

### Additional biochemical parameters and side effects

Table 3 shows data on albuminuria, volume status, and plasma and urine measurements at baseline and after treatment with DRI and ACEi. Urinary albumin excretion rate was in the normal range at baseline (20 [9–42] mg/day) and showed a significant reduction by DRI (12 [5–28] mg/day, P = 0.030), however not by ACEi (16 [7–35] mg/day, P = 0.3). Urinary protein excretion rate was in the low-normal range at baseline (0.1 [0.1–0.1] g/day), however, unresponsive to either DRI or ACEi.

**Table 3:** Clinical parameters at baseline and after treatment with ACEi and DRI.

	Baseline	ACEi	DRI
**Clinical parameters**			
Body weight (Kg)	101 (3)	100 (3)	100 (3)
ECV (L)	22.7 (1.1)	23.8 (1.3)	24.1 (1.0)
**Plasma and serum measurements**			
Hemoglobin (mmol/L)	9.5 (0.2)	9.5 (0.1)	9.4 (0.2)
Hematocrit (v/v)	0.45 (0.01)	0.45 (0.01)	0.44 (0.01)
Sodium (mmol/L)	141 (0.4)	141 (0.4)	140 (0.5)
Potassium (mmol/L)	3.9 (0.1)	3.9 (0.1)	4.0 (0.1)*
Urea (mmol/L)	6.1 (0.3)	5.9 (0.3)	5.7 (0.3)
Creatinine (μmol/L)	78 (3)	79 (3)	77 (3)
Creatinine clearance (mL/min)	141 (7)	147 (13)	140 (12)
**Urine measurements**			
Albuminuria (mg/day)	20 [9–42]	16 [7–35]	12 [5–28]*
Proteinuria (g/day)	0.1 [0.1–0.1]	0.1 [0.1–0.1]	0.1 [0.1–0.1]
Urinary volume (mL/day)	2106 (195)	2076 (161)	2178 (193)
Creatinine excretion (mmol/day)	15.9 (0.7)	16.9 (1.0)	16.1 (0.6)
Sodium excretion (mmol/day)	205 (21)	248 (16)	223 (16)

Data are shown as mean (SEM) or as geometric mean (95% CI) when indicated.

* P<0.05 vs. baseline.

** P<0.01 vs. baseline.

Mean urinary sodium excretion at baseline was 205 (21) mmol/day and remained stable throughout the trial. In line with this, ECV, body weight, and urinary volume remained also unaffected by either DRI or ACEi. Serum potassium at baseline (3.9 (0.1) mmol/L) showed a small but significant increase by DRI (4.0 (0.1) mmol/L, P = 0.043), but not by ACEi (4.0 (0.1) mmol/L, P = 0.5). None of the subjects developed hyperkalemia (defined as K^+^>5.0 mmol/L). Urinary potassium excretion remained stable throughout the trial.

One patient complained of dry cough and symptomatic hypotension during treatment with DRI, which did not result in dosage reduction of trial medication and was resolved after completion of the trial period.

## Discussion

In men with weight excess and hypertension, DRI significantly increased ERPF, and reduced RVR and FF, along with a significant reduction in systemic blood pressure and albuminuria. ACEi, which served as a positive control, significantly increased ERPF and reduced RVR, with a nominal reduction in FF. RAAS activity was significantly reduced by both DRI and ACEi. This trial demonstrated that DRI is an effective treatment for obese hypertensive men, in line with the known beneficial effect of ACEi, with a potentially favorable renal profile.

The renal hemodynamic response to DRI, as found in this trial, is in line with previous studies in essential hypertensives during liberal sodium intake [18,25–29]. Studies that specifically investigated aliskiren also found a renal vasodilator response [24,30]. Of note, these studies included normal weight subjects only, whereas overweight and obese subjects could theoretically benefit most from direct renin blockade. Our trial therefore adds to the current literature as it is the first to demonstrate an effect of DRI on renal hemodynamics in subjects with weight excess and hypertension.

We found a significant reduction in automatic twenty-four hour ambulatory and semi-automatic office blood pressure. In contrast to renal hemodynamics, the effect of DRI on systemic hemodynamics has been relatively well characterized in obese subjects, with studies reporting of an unequivocal reduction in blood pressure [31–37], that sustained over a minimal period of 6 to 12 months [33,36,37].

Furthermore, we found a pronounced reduction in RAAS activity in response to DRI, as reflected by a significant reduction in plasma renin activity and a significant increase in both plasma renin concentration and urinary renin excretion. Moreover, we found a significant reduction in plasma and urinary aldosterone/renin concentration ratio in response to DRI, accompanied by a significant reduction in urine/plasma concentration ratio of renin. The rise in renin excretion combined with the reduction in the urine/plasma concentration ratio of renin raises the question to what degree these data indicate activation or suppression of the renal RAAS. Here it is important to realize that the majority of urinary renin is plasma-derived. This is evidenced by the up to 40-fold higher urinary renin levels in patients with Dent’s disease or Lowe syndrome, in whom tubular reabsorption of filtered renin (by megalin) is disturbed [38]. On this basis, urinary renin levels should be corrected for plasma renin levels. Yet, there is also evidence for renin production in the collecting duct. Surprisingly, here angiotensin II stimulates local renin release, as opposed to its inhibitory effects on renin release in the juxtaglomerular apparatus [39,40]. Therefore, given the drop in Angiotensin II after DRI, and assuming that some urinary renin originates from the collecting duct, a decrease in the urine/plasma concentration ratio of renin would be expected. Since this is exactly what occurred, it appears that DRI attenuates RAAS activation in the kidney, i.e., does not result in selective upregulation of collecting duct renin. Clearly however, alternative explanations, like alterations in tubular megalin expression, should be considered as well.

Another question that needs to be addressed is to what degree the elevated renin levels during DRI would exert angiotensin II-independent effects, via binding to the so-called (pro)renin receptor [41]. Indeed, in vitro, renin inhibitors do not interfere with such binding [42], although obviously they do block renin activity. Renin/prorenin binding to this receptor results in the activation of multiple second messenger pathways [41]. However, the binding affinity of this receptor for renin and its precursor prorenin was found to be in the nanomolar range, while their in-vivo levels are many orders below this range, even during DRI treatment [43, 44]. On this basis, combined with the fact that the (pro)renin receptor is now additionally known to be an accessory subunit of vacuolar H^+^-ATPase, displaying multiple effects that are entirely unrelated to renin and/or prorenin, e.g., with regard to cardiomyocyte autophagy [45], polyuria and renal acid-base regulation [46], and LDL metabolism [47], this idea is now being abandoned [48].

Aldosterone levels were significantly reduced by DRI in urine, however, not in plasma, despite the fact that urinary aldosterone is plasma-derived. This is most likely related to much lower concentrations in plasma compared to urine, which limits the ability to detect statistical differences. Our observations are in line with studies stating that plasma aldosterone is a less sensitive marker of RAAS activity [49]. We found a reduction in urinary angiotensinogen which was paralleled by a reduction in urine/plasma ratio of angiotensinogen. Urinary angiotensinogen might be both kidney- and plasma-derived. In case of the former, the reduction in urinary angiotensinogen would be suggestive a for a suppression of renal RAAS activity [50]. In case of the latter, angiotensinogen would be a marker for permeability of the glomerular filtration barrier [49, 51]. Our data, showing a similar response of urinary angiotensinogen and albuminuria to DRI, are consistent with the second concept. Moreover, 2 seminal papers by Matsusaka et al. [52, 53], making use of kidney-specific angiotensinogen knockout mice, revealed that, both under normal and pathological conditions, renal angiotensin II production depends entirely on plasma-derived (i.e., hepatic) angiotensinogen. Therefore, the function of locally produced angiotensinogen in the kidney, if any, remains controversial.

We believe that our trial adds to the current understanding of the RAAS in response to DRI by its extensive characterization of multiple RAAS parameters simultaneously, in both plasma and urine, thereby rendering an effect of DRI not only on systemic, but also on intrarenal RAAS activity plausible [54,55].

DRI might have a stronger effect on renal hemodynamics than other RAAS blocking agents [24,55], with possibly a stronger effect on systemic hemodynamics as well [34–36, 54]. One explanation for this could be that RAAS activity is more efficiently blocked by DRI, as it intervenes in the RAAS at its point of activation [56]. Another explanation might be its long pharmacokinetic half-life (up to 36 hours [56] and/or its ability to penetrate adipose, skeletal and renal tissue—with renal accumulation of aliskiren up to two weeks after drug withdrawal—thereby affecting RAAS activity at a tissue level [57, 58]. In our trial, we found only a trend towards stronger effect of DRI, although it should be noted that our trial was not designed nor powered to investigate a quantitative difference in efficacy between DRI and ACEi. A quantitative difference in efficacy can only be adequately investigated in a dose-response study.

Several limitations of this trial should be considered. First of all, we studied the effects of DRI during liberal sodium intake, while the effect of RAAS blockade is known to be potentiated by even mild sodium restriction, or diuretics [59]. The efficacy in a setting of clinical treatment of hypertension might thus been underestimated. Second, as the number of studied subjects was relatively small, we optimized the signal-to-noise ratio by solely including men, as physiological variation in circulating estrogens levels in women is known to influence renal hemodynamic measurements and RAAS activity [60,61]. Whereas the cross-over design of the trial, with every subject being its own control, provided us adequate power to detect effects of DRI, however, this set-up limits the generalizability of the results. Third, we did not include a control group to compare the renal response to DRI between healthy and obese subjects.

In conclusion, we found a favorable renal and systemic hemodynamic response to DRI, accompanied by a reduction in RAAS activity and albuminuria, in men with weight excess and hypertension.

## Supporting Information

S1 CONSORT Checklist (DOC)

S1 Text Trial protocol. (PDF)

S1 Table Subject characteristics stratified by treatment of initiation. (DOCX)
