# Supplementary material for: Effects of Direct Renin Blockade on Renal & Systemic Hemodynamics and on RAAS Activity, in Weight Excess and Hypertension: A Randomized Clinical Trial
Source: PLoS One. 2017 Jan 24;12(1):e0169258. doi: 10.1371/journal.pone.0169258 (PMC5261569; doi:10.1371/journal.pone.0169258)
Supplement: S1 Text — (PDF) [file pone.0169258.s002.pdf]

Renal hemodynamic effects of aliskiren in comparison to ramipril

The renal HEALTH-STudY

Renal Hemodynamic Effects of ALiskiren (rasilez) in comparison to ramipril (Tritrace) in patients with overweigHt/obeSiTy and hYpertension

PROTOCOL TITLE: Renal Hemodynamic Effects of ALiskiren (rasilez) in comparison to ramipril (Tritrace) in patients with overweigHt/obeSiTy and hYpertension: The renal HEALTH-STudY

**Protocol ID**	**…**
**Short title**	**Renal hemodynamic effects of aliskiren in comparison of ramipril**
**Version**	**2.0**
**Date**	**September 2010**
**Coordinating investigators**	**drs. A.J. Kwakernaak (M) / dr. H.J. Lambers-Heerspink (M)**<br>**University Medical Center Groningen (UMCG), Department of Medicine, Division of Nephrology / Faculty of Medicine, Department of Clinical Pharmacology**<br>**“De Brug”, room 4.045**<br>**P.O. Box 30.001, 9700 RB Groningen, the Netherlands**<br>**T: +31 50 361 1564**<br>**E: a.kwakernaak@int.umcg.nl**
**Principal investigator**	**prof. dr. G.J. Navis (V) / dr. G.D. Laverman (M)**<br>**University Medical Center Groningen (UMCG),**<br>**Department of Medicine, Division of Nephrology**<br>**De Brug, room 4.050 / 4.044**<br>**P.O. Box 30.001, 9700 RB Groningen, the Netherlands**<br>**T: +31 50 361 4441 / +31 50 361 5120**<br>**E: g.j.navis@int.umcg.nl / g.d.laverman@int.umcg.nl**
**Sponsor**	**University Medical Center Groningen**
**Independent physician**	**dr. C.F.M. Franssen**<br>**University Medical Center Groningen (UMCG),**

	**Department of Medicine, Division of Nephrology**<br>**De Brug, kamer 4.041**<br>**P.O. Box 30.001, 9700 RB Groningen, the Netherlands**<br>**T: +31 50 361 3415**<br>**E: c.f.m.franssen@int.umcg.nl**
**Laboratory site**	**Clinical Chemical Laboratory**<br>**University Medical Center Groningen (UMCG)**<br>**Hanzeplein 1, 9700 RB Groningen, the Netherlands**<br>**T: +31 50 361 1840**
**Pharmacy**	**Clinical Pharmacy**<br>**University Medical Center Groningen (UMCG)**<br>**Hanzeplein 1, 9700 RB Groningen, the Netherlands**<br>**T: +31 50 361 4331**

PROTOCOL SIGNATURE SHEET

Name	Signature	Date
**Head of Department:**<br><br>***Prof. dr. P.E. de Jong***		
**Principal Investigator:**<br><br>***Mw. prof. dr. G.J. Navis***		

TABLE OF CONTENTS

1. INTRODUCTION AND RATIONALE…………………………………………………………...10
2. OBJECTIVES 11
3. STUDY DESIGN 12
4. STUDY POPULATION 14
4.1 Population (base) 14
4.2 Inclusion criteria 14
4.3 Exclusion criteria 5
4.4 Sample size calculation 15
5. TREATMENT OF SUBJECTS 16
5.1 Investigational product 16
5.2 Use of co-intervention 16
5.3 Escape medication 17
6 INVESTIGATIONAL MEDICINAL PRODUCT 18
6.3 Name and description of investigational medicinal product 18
6.4 Summary of findings from non-clinical studies 19
6.5 Summary of findings from clinical studies 19
6.6 Summary of known and potential risks and benefits 20
6.7 Description and justification of route of administration and dosage 20
6.8 Dosages, dosage modifications and method of administration 20
6.9 Preparation and labelling of Investigational Medicinal Product 20
6.10 Drug accountability 21
7 METHODS 22
7.3 Study parameters/endpoints 22
7.3.1 Main study parameter/endpoint 22
7.3.2 Secondary study parameters 22
7.3.3 Other study parameters 22
7.4 Randomisation, blinding and treatment allocation 22
7.5 Study procedures 22
7.6 Withdrawal of individual subjects 23
7.6.1 Specific criteria for withdrawal 24
7.7 Replacement of individual subjects after withdrawal 24
7.8 Follow-up of subjects withdrawn from treatment 25
7.9 Premature termination of the study 25
8 SAFETY REPORTING 26
8.1 Section 10 WMO event 26
8.2 Adverse and serious adverse events 26
8.2.1 Suspected unexpected serious adverse reactions (SUSAR) 26
8.2.2 Annual safety report 28
8.3 Follow-up of adverse events 28
8.4 Data Safety Monitoring Board (DSMB) 28
9 STATISTICAL ANALYSIS 29
9.1 Descriptive statistics 29

9.2 Univariate analysis....29
9.3 Multivariate analysis....29
9.4 Interim analysis....29
10 ETHICAL CONSIDERATIONS....30
10.1 Regulation statement....30
10.2 Recruitment and consent....30
10.3 Objection by minors or incapacitated subjects....30
10.4 Benefits and risks assessment, group relatedness....30
10.5 Compensation for injury....31
10.6 Incentives....31
11 ADMINISTRATIVE ASPECTS AND PUBLICATION....32
11.1 Handling and storage of data and documents....32
11.2 Amendment....32
11.3 Annual progress report....32
11.4 End of study report....32
11.5 Public disclosure and publication policy....33
12 REFERENCES....34

LIST OF ABBREVIATIONS AND RELEVANT DEFINITIONS

**ABR**	**ABR form (General Assessment and Registration form) is the application form that is required for submission to the accredited Ethics Committee (ABR = Algemene Beoordeling en Registratie)**
**AE**	**Adverse Event**
**AR**	**Adverse Reaction**
**CA**	**Competent Authority**
**CCMO**	**Central Committee on Research Involving Human Subjects**
**CV**	**Curriculum Vitae**
**DSMB**	**Data Safety Monitoring Board**
**EU**	**European Union**
**EudraCT**	**European drug regulatory affairs Clinical Trials GCP Good Clinical Practice**
**IB**	**Investigator's Brochure**
**IC**	**Informed Consent**
**IMP**	**Investigational Medicinal Product**
**IMPD**	**Investigational Medicinal Product Dossier**
**METC**	**Medical research ethics committee (MREC); in Dutch: medisch ethische toetsing commissie (METC)**
**(S)AE**	**Serious Adverse Event**
**SPC**	**Summary of Product Characteristics (in Dutch: officiële productinfomatie IB1-tekst)**
**Sponsor**	**The sponsor is the party that commissions the organisation or performance of the research, for example a pharmaceutical company, academic hospital, scientific organisation or investigator. A party that provides funding for a study but does not commission it is not regarded as the sponsor, but referred to as a subsidising party.**
**SUSAR**	**Suspected Unexpected Serious Adverse Reaction**
**Wbp**	**Personal Data Protection Act (in Dutch: Wet Bescherming Persoonsgevens)**
**WMO**	**Medical Research Involving Human Subjects Act (Wet Medisch-wetenschappelijk Onderzoek met Mensen**

SUMMARY

Rationale

The combination of hypertension and overweight/obesity is a highly prevalent clustering of cardiovascular risk factors which significantly contributes to over-all morbidity and mortality. In overweight/obese and hypertensive patients, increased glomerular pressure is considered to be a driving force for progressive end-organ damage of the kidney. This increased glomerular pressure is associated with an increased intrarenal RAAS-activity and can be inhibited by RAAS blocking drugs (ACEi, ARB). Recently, a new class of direct renin inhibitors has been developed which particularly effective inhibits intrarenal RAAS and therefore might be more potent than ACEi and ARB. However, the effect of this new class of direct renin inhibitors on glomerular pressure, independent from blood pressure, has not yet been studied.

Objective

To determine the effects of aliskiren, as compared to ramipril, on renal hemodynamics in overweight/obese and hypertensive patients.

Study design

The study is designed as a single-centre, double-blind, double-dummy, cross-over, randomized clinical trial.

Study population

The study population consists of 16 non-diabetic male patients with overweight/obesity (BMI ≥27 and ≤35 kg/m^2) and essential hypertension according to WHO-criteria (systolic and diastolic blood pressure ≥140 or ≥90 mmHg, respectively) in the age of 18 - 70 years. All patients have normal renal function (creatinine clearance >90 ml/min/1,73m^2) and normo- or microalbuminuria (albuminuria ≤300mg/day).

Intervention

After a wash-out period of 6 weeks, patients are randomly assigned to either a 6-week treatment period with aliskiren or a 6-week treatment period with ramipril in a cross-over design. Between both treatment periods an 8-week wash-out period is present.

Primary study parameters/endpoints

The main study parameters/endpoints are:

- Renal hemodynamics (GFR, ERPF, FF)
- Blood pressure

Secondary study parameters/endpoints

- Volume status (extracellular fluid volume - ECV)
- RAAS parameters (plasma renin activity, plasma renin concentration, angiotensin II, aldosteron)
- Urinary and serum kidney injury markers, such as, but not limited to, TGFß, KIM-1, NGAL, NAG, uromodulin and prostasin.

Other study parameters

- routine blood biochemistry (renal function, electrolytes, liver enzymes, lipid profile, glucose, volume parameters) and full blood cell count
- routine urinary chemistry (creatinine, albumin urea, potassium and sodium)

Nature and extent of the burden and risks associated with participation, benefit and group relatedness

Patients visit the outpatient nephrology clinic on a more regular base than standard patient care - i.e. at study inclusion and at start and end of each treatment period (5 hospital visits in a total study duration of 26 weeks) - for clinical assessment, renal hemodynamics (GFR, ERPF, FF) volume status (ECV) measurements. Renal hemodynamics are measured by infusion of radioactive isotopes (GFR by iothalamate; ERPF by hippuran and FF as a ratio of GFR and ERPF) and volume status is calculated using the distribution volume of iothalamate. A fasting blood sample is collected with a venapuncture already performed for measurement of renal hemodynamics. Blood pressure is measured by ambulant device (Spacelab) and 24hr urine is collected at the day prior to the hospital visit. The amount of radioactive radiation during renal hemodynamic measurements is comparable to a single X-thorax (16% of the yearly dosage of background radiation in the Netherlands). In this hospital, renal hemodynamic measurements are routinely performed for daily patient care and are of minimal risk. No other invasive measurements will be executed. Patients receive financial support of 200 euro and restitution of all travel costs. Patients receive no priority in treatment of other diseases in the clinic during this study. There are no direct benefits for the patients to be included and participation is on a free-will base.

1. INTRODUCTION AND RATIONALE

The combination of hypertension and obesity is a highly prevalent clustering of cardiovascular risk factors which considerably contributes to over-all morbidity and mortality in the general population. The kidneys play a central role in the interaction between these two risk factors.

Obese or overweight hypertensive patients have increased glomerular pressure (1), which is considered a driving force for progressive end-organ damage of the kidney (2), thus contributing to the vicious circle of hypertension and target organ damage. In line, weight excess is an independent risk factor for renal damage that adds to the effect of elevated blood pressure (3;4). Accordingly, specific correction of increased glomerular pressure, on top of reduction of systemic blood pressure, has been shown to be renoprotective in experimental animals and in renal patients (5;6). Glomerular pressure is determined by the net balance between the tone of the afferent and efferent glomerular vessels and is mainly regulated by the Renin Angiotensin Aldosterone System (RAAS) that modifies efferent vascular tone. This is particularly relevant given the availability of antihypertensive drugs that specifically interfere in the RAAS, i.e. the ACE-inhibitors (ACEi), the Angiotensin Receptor Blockers (ARB) and specific renin-inhibition. Based on these considerations it is likely that the renal hemodynamic actions of RAAS-blockade will be of specific benefit in overweight or obese hypertensive patients.

Aliskiren represents the new class of direct renin inhibitors, i.e. blockade of the RAAS at its point of activation. Importantly, the effects of aliskiren on glomerular pressure have not been tested so far. A beneficial short term (decrease of glomerular pressure) and long term (prevention of end stage renal disease) renal profile has been proven with the older RAAS blocking drug classes (ACEi, ARB) (7). It seems logical to assume that aliskiren will decrease glomerular pressure as well, in particular because renin-inhibition with older renin-inhibitors was shown to have more potent renal effects than ACEi and ARB. In our hands renin-inhibition with remikiren was shown to reduce glomerular pressure in hypertensive and normotensive volunteers (8).

2. OBJECTIVES

Primary Objective:

The primary objective of this study is to determine the effects of aliskiren, as compared to ramipril, on renal hemodynamics in overweight/obese and hypertensive patients.

Research question:

Does aliskiren ameliorate glomerular hypertension in overweight/obese hypertensive patients, in addition to its effect on blood pressure?

3. STUDY DESIGN

The study question will be addressed in a prospective, single-centre, double-blind, double-dummy, cross-over, randomized clinical trial. Before inclusion, patients visit the outpatient nephrology clinic for clinical assessment. Patients treated with antihypertensive medication will enroll in a 6 week wash-out period in which antihypertensive medication is discontinued. In week 3 of the wash-out period, systolic and diastolic blood pressure is measured and must remain lower than 180 and 110 mmHg, respectively. When blood pressure exceeds these values, the patient will be considered a screen failure and prior antihypertensive medication is restarted. A wash-out period is not mandatory in patients with no antihypertensive medication. Consecutively, patients will be randomly assigned to either a 6-week treatment period with aliskiren and ramipril-placebo or a 6-week treatment period with ramipril and aliskiren-placebo, in a cross-over design. To safeguard patients, serum potassium and blood pressure is measured 2 weeks after start of each treatment period (see paragraph 7.4.1 for action to be taken when hyperkalemia or hypotension occurs). Between both treatment periods an 8-week wash-out period is present in which systolic and diastolic blood pressure are measured and must remain lower than 180 and 110 mmHg, respectively. When blood pressure exceeds these values, clonidin is started in an initial dosage of 2 dd 0.075 mg with subsequent increase of dosage (maximal 3 dd 0.15 mg) to reach target blood pressure. Patients visit the outpatient nephrology clinic at start and end of both treatment periods (four times in total) for clinical assessment and renal hemodynamic (GFR, ERPF and FF) and volume status (ECV) measurements. Renal hemodynamics are measured by infusion of radioactive isotopes (GFR by iothalamate; ERPF by hippuran and FF as a ratio of GFR and ERPF) and volume status (ECV) is calculated using the distribution volume of iothalamate. A fasting blood sample for measurement of status of the renin-angiotensin-aldosterone system, including plasma renin activity, plasma renin concentration, angiotensin II, aldosterone and biomarkers, is collected with a venapuncture already performed for measurement of renal hemodynamics. Urine is also collected for measurement of biomarkers. Additional parameters include routine blood and urine chemistry and total blood cell count. Blood pressure is measured by an ambulant device (Spacelab) and 24hr urine is collected at the day prior to the hospital visit.

Patients will be instructed to take the study medication once daily, in the morning, except on study days; on those days, the study drug will not taken before renal hemodynamic measurements at the hospital (between 8:00 and 9:30 a.m.). In total, patients visit the outpatient nephrology clinic on a more regular basis than standard patient care (5 hospital visits in a total study duration of 26 weeks).

In summary, this is the study protocol (with treatment sequence randomized):

6 weeks	6 weeks	8 weeks	6 weeks
wash-out period	**aliskiren (rasilez) & ramipril placebo**<br><br><u>double-dummy</u>	**wash-out period**	**ramipril (tritrace) & aliskiren placebo**<br><br><u>double-dummy</u>
blood pressure monitoring at week 3	blood pressure and serum potassium monitoring at week 2	blood pressure monitoring at week 3	blood pressure and serum potassium monitoring at week 2

- clinical assessment
- renal hemodynamics and volume status measurement
- blood pressure measurement
- 24hr urine collection

- clinical assessment
- renal hemodynamics and volume status measurement
- blood pressure measurement
- 24hr urine collection

4. STUDY POPULATION

4.1 Population (base)

The study population will be selected from the outpatient clinic of the department of internal medicine of the University Medical Centre Groningen (UMCG) and from clinics of specifically selected general practitioners.

4.2 Inclusion criteria

- Male caucasian patients
- Age ≥18 and ≤70 years
- Overweight or obese (BMI ≥27 and ≤35 kg/m^2)
- Essential hypertension according to WHO-criteria (systolic and diastolic blood pressure ≥140 or ≥90 mmHg, respectively)
- Normal renal function (creatinine clearance >90 ml/min/1.73m^2)
- Normo- or microalbuminuria (albuminuria ≤300mg/day)
- Written informed consent

4.3 Exclusion criteria (adapted from ARIA-study: METc 2010.114)

- Inability to meet inclusion criteria
- Previously treated (within 3 months prior to start of study) with aliskiren or ramipril.
- Cardiovascular disease (myocardial infarction, angina pectoris, percutanous transluminal coronary angioplasty, coronary artery bypass grafting, stroke, heart failure (NYHA I-IV), Diabetes Mellitus
- Active malignancy
- Any medication, surgical or medical condition which might significantly alter the absorption, distribution, metabolism, or excretion of medications including, but not limited to any of the following:
    - History of active inflammatory bowel disease within the last six months;
    - Major gastrointestinal tract surgery such as gastrectomy, gastroenterostomy, or bowel resection;
    - Gastro-intestinal ulcers and/or gastrointestinal or rectal bleeding within last six months;
    - Pancreatic injury or pancreatitis within the last six months;
    - Evidence of hepatic disease as determined by any one of the following: ALT or AST values exceeding 3x ULN at inclusion, a history of hepatic

encephalopathy, a history of esophageal varices, or a history of portocaval shunt;
  - Evidence of urinary obstruction of difficulty in voiding at inclusion

- History of severe hypersensitivity or contraindications to ramipril or aliskiren
- Hypersensitivity to ^{125}I-iothalamate or ^{131}I-hippuran
- History of angioedema
- History of autonomic dysfunction (e.g. history of fainting or clinically significant orthostatic hypotension)
- Participation in any clinical investigation within 3 months prior to start of the study
- Donation or loss of 400 ml or more of blood within 3 months prior to initial dosing
- History of drug or alcohol abuse within the 12 months prior to dosing, or evidence of such abuse as indicated by the laboratory assays conducted during the screening.
- History of noncompliance to medical regimens or unwillingness to comply with the study protocol.
- Any surgical or medical condition, which in the opinion of the investigator, may place the patient at higher risk from his/her participation in the study, or is likely to prevent the patient from complying with the requirements of the study or completing the study.

4.4 Sample size calculation

We hypothesized that patients will present a mean±SD ERPF of 444±35 ml/in at baseline (i.e. after discontinuation of antihypertensive medication). Assuming an increase of 13±11 ml/min with ACEi (10) and an increase of 47±17 ml/min with aliskiren (11), it was estimated that 14 patients had to complete the cross-over design sequence to give the study a 90% power to detect a statistically significant difference (α=0.05). On the basis of an expected dropout rate of 10% due to the possibility of side effects in patients on aliskiren or ramipril, 16 patients will be included in this study.

5. TREATMENT OF SUBJECTS

5.1 Investigational product

Aliskiren, ramipril tablets and matching placebo's (in a double-blind, double-dummy design) are provided by Novartis. Patients take 10 mg ramipril (once daily) or 300 mg aliskiren (once daily) according to randomised treatment scheme. Study medication is received at the study site by a designated person, handled and stored safely and properly, and kept in a secured location. The study medication is stored according to the instructions specified on the drug labels. Storage conditions are adequately monitored. Subjects are asked to return all unused study drug and packaging at the end of the study or at the time of study drug discontinuation or in every visit to the outpatient nephrology clinic. Appropriate documentation of the subject specific dispensing process is maintained. Unused drugs are destroyed by the pharmacy department at the end of the study.

5.2 Use of co-intervention (criteria adapted from ARIA-study: METc 2010.114)

Use of the following treatments is NOT allowed after the start of the study as these medications may interfere with the evaluation of safety, tolerability and/or efficacy. Patients who are receiving such medication(s) should be excluded, or if ethically justified, the medication(s) may be withdrawn according to the manufacturer's/investigator's instructions prior to start of the screening period. Withdrawal of medication is done after consultation with the patient and patients' physician

- RAAS-medication, diuretics or ß-blockers
  - NSAIDS/COX-2 inhibitors. Continuous use is not allowed during the trial. Acute and intermittent use of acetaminophen and aspirin is permitted throughout the trial
  - Oral corticosteroids. Inhaled, topical and nasal corticosteroid preparations may be used as needed
  - Antipsychotic agents (e.g., clozapine, haloperidol or phenothiazines)
  - Thyroid medication and/or low dose hormone replacement therapy, and statins unless these have been stable maintenance doses for 3 months prior to study
  - Chronic administration (defined as administration >3 days per week) of sympathomimetic drugs such as those found in nasal decongestants (e.g., pseudoephedrine, phenylpropanolamine) and bronchodilators (e.g., metaproterenol). Occasional use of these drugs (if not within 48 hours of a scheduled visit) is allowed

- Ergot and serotonin (5-hydroxytryptamine) receptor agonists (e.g., sumatriptan).
- Phosphodiesterase type 5 (PDE5) inhibitors such as sildenafil (Viagra), vardenafil (Levitra),and tadalafil (Cialis) are not allowed within 48 hours prior to any scheduled visit
- Antiarrhythmic drugs including digoxin
- Cholestyramine and colestipol resins
- Potent and moderate PgP inhibitors for example cyclosporine. For moderate PgP inhibitors such as ketoconazole and verapamil, caution should be exercised

5.3 Escape medication

- Clonidin (maximum dosage of 3 dd 0.15 mg)

6 INVESTIGATIONAL MEDICINAL PRODUCT

6.1 Name and description of investigational medicinal product

Ramipril (tritace) is an inhibitor of the angiotensin converting enzyme (ACE) which converts angiotensin I to angiotensin II. The levels of angiotensin I and II decrease and the plasma renin activity increases.

Following oral administration ramipril is rapidly absorbed from the gastrointestinal tract; peak plasma concentrations of ramipril are reached within one hour. Peak plasma concentrations of the active metabolite, ramiprilat, are reached within 2 - 4 hours. Plasma concentrations of ramiprilat decline in a polyphasic manner. The effective half-life of ramiprilat after multiple once daily administration of ramipril is 13 - 17 hours for 5 -10 mg ramipril and markedly longer for lower doses, 1.25 - 2.5 mg ramipril. This difference is related to the long terminal phase of the ramiprilat concentration time curve observed at very low plasma concentrations. This terminal phase is independent of the dose, indicating a saturable capacity of the enzyme to bind ramiprilat. Steady-state plasma concentrations of ramiprilat after once daily dosing with the usual doses of ramipril are reached by about the fourth day of treatment. Ramipril is almost completely metabolized and the metabolites are excreted mainly via the kidneys. In addition to the bioactive metabolite, ramiprilat, other, inactive metabolites have been identified, including diketopiperazine ester, diketopiperazine acid and conjugates.

Aliskiren (rasilez) is a selective inhibitor of the enzyme renin and thereby targeting the conversion of angiotensinogen to angiotensin I, resulting in decreased levels of angiotensin I, II and renin. Aliskiren thereby lowers systolic and diastolic blood pressure.

The single and multiple-dose oral pharmacokinetics of aliskiren has been investigated over the dose range 40 to 1800 mg (aliskiren hemifumarate) in healthy subjects and in patients. Maximum plasma concentrations of aliskiren were observed between 2 and 4 h post-dose (median tmax). Half life ranged from 34 to 41 hours. Mean absolute bioavailability was 2.6% (for a 75 mg hard gelatin tablet formulation). Administration of aliskiren together with food decreased mean Cmax and AUC0-inf by 81 and 62%, respectively. After multiple dosing, the mean terminal half-life was between 23 and 36 h. Excretion of unchanged aliskiren in urine was 0.11 to 0.45%. No significant interaction has been found with co-administration of irbesartan, furosemide, digoxin, ramipril and hydrochlorothiazide. Co-administration of metformin or amlodipine resulted in less than 30% change in Cmax or AUC of aliskiren. When aliskiren was co-administered with furosemide, the AUC and Cmax of furosemide were reduced by 28% and 49%

respectively. Co-administration of ketoconazole with aliskiren resulted in increased plasma levels of aliskiren (AUC and Cmax by 76% and 81% respectively). Co-administration of atorvastatin with aliskiren had no effect on the steady-state pharmacokinetics of atorvastatin or its two active metabolites, but there was an increase in steady-state Cmax and AUC by approximately 50%. Studies were also performed with cimetidine, atenolol, acenocoumarol and celecoxib. There were no clinically relevant changes in the pharmacokinetic parameters of any of these drugs, or aliskiren, when combined or when given alone. Aliskiren should not be given together with strong PgP inhibitors like cyclosporin.

6.2 Summary of findings from non-clinical studies

This is not applicable as ramipril and aliskiren are both registered for treatment of blood pressure and progression of renal disease in humans.

6.3 Summary of findings from clinical studies

Aliskiren inhibits the RAAS in a dose dependent manner. The evidence for RAAS blockade is based on the effects of aliskiren on plasma renin activity, angiotensin I and II levels, urinary aldosterone levels and plasma rennin concentration. Plasma PRA is a direct measure of the formation of Ang I in the plasma. Plasma renin concentration (PRC), measures the concentration of immunoactive renin in the plasma. Phase 2 and 3 clinical studies involving over 9000 subjects with hypertension have demonstrated that aliskiren provides effective long-term BP lowering with a good safety and tolerability profile at does of 150 and 300 mg/day. Aliskiren inhibits PRA by up to 80% following both single and multiple oral dose administration. Similar reductions in PRA are observed when aliskiren is administered in combination with agents that increase PRA, including diuretics, ACEi and ARB's, despite greater increases in PRC. Moreover, PRA inhibition and BP reductions persist for 2 to 4 weeks after stopping aliskiren treatment.

After 8 weeks of treatment of diabetic patients with hypertension [CSPP100A2307], PRA was significantly increased from baseline with ramipril monotherapy (10 mg, 106%; $p<0.001$ vs baseline). In contrast, aliskiren monotherapy produced a statistically significant reduction from baseline in PRA of 66% (300 mg, $p<0.001$). Aliskiren-based therapy also reduced PRA despite causing a rise in plasma renin concentration [CSPP100A2306]. At the end of the 26-week treatment of mild to moderate hypertension patients, aliskiren-based therapy (aliskiren 150 or 300 mg) reduced PRA by 63% (relative to baseline), whereas ramipril-based therapy (ramipril 5 or 10 mg) increased PRA by 143%.

6.4 Summary of known and potential risks

Ramipril

Ramipril is approved for the treatment of hypertension by EMEA, FDA and Dutch registration board (CVZ). Generally, adverse reactions with ramipril (tritace) have been mild and transient, and did not require discontinuation of therapy. The most frequently reported adverse reactions are nausea, dizziness and headache. Uncommonly drowsiness, light headedness or impaired reactions may occur. Reactions such as peripheral edema, tinnitus, fatigue, visual disturbances, sweating, disturbed hearing, and disturbed orthostatic regulation are rare. Symptomatic hypotension accompanied by dizziness, weakness and nausea may occur after the initial dose of Ramipril and after an increase in the dose of ramipril. It has been rarely observed, but may occur in severely salt/volume-depleted patients. Ramipril can result in hyperkalemia. Data on pharmacokinetics are given in paragraph 6.1.

Aliskiren

Aliskiren (rasilez) has been approved by EMEA, FDA and Dutch registration board (CVZ) for and has been prescribed in clinical setting to treat hypertension and progression of renal disease. No serious side effects are expected at the dosage used in this study. Possible side effects of aliskiren are orthostatic hypotension, diarrhoea and rash. Aliskiren can result in hyperkalemia. Data on pharmacokinetics are given in paragraph 6.1.

6.5 Description and justification of route of administration and dosage

Aliskiren and ramipril are fully absorbed from the digestive tract and can therefore be orally administered.

6.6 Dosages, dosage modifications and method of administration

Aliskiren (tablets): 300 mg once daily, orally.

Ramipril (tablets): 10 mg once daily, orally.

6.7 Preparation and labelling of Investigational Medicinal Product

Study medication (aliskiren, ramipril and matching placebo's) will be provided by Novartis and repacked and labelled by the pharmacy unit of the University Medical Center Groningen (UMCG).

6.8 Drug accountability

All study medication will be stored at room temperature at the pharmacy department of the University Medical Center Groningen (UMCG). Study medication will be collected from the pharmacy department by the coordinating investigator once a week. Non-used medication will be returned to the pharmacy department where it will be destructed.

7 METHODS

7.1 Study parameters/endpoints

7.1.1 Primary study parameters

- Renal hemodynamics (GFR, ERPF, FF)
- Blood pressure

7.1.2 Secondary study parameters

- Volume status (ECV)
- RAAS parameters (plasma renin activity, plasma renin concentration, angiotensin II, aldosteron)
- Urinary and serum kidney injury markers, such as, but not limited to, TGFß, KIM-1, NGAL and NAG.

7.1.3 Other study parameters

- routine blood biochemistry (renal function, electrolytes, liver enzymes, lipid profile, glucose, volume parameters) and full blood cell count
- routine urinary chemistry (creatinine, albumin, urea, potassium and sodium)

7.2 Randomisation, blinding and treatment allocation

Treatment sequence of aliskiren and ramipril will be randomized by computer randomization with a batch size of 2. Patients are treated double-dummy.

7.3 Study procedures

<u>Physical examination</u>

Patients will be subjected to physical examination by the investigator at study inclusion. This physical examination entails a routine investigation of heart, lungs and abdomen.

<u>Renal hemodynamic measurements</u>

At start and end of each treatment period, glomerular filtration rate (GFR) and effective renal plasma flow (ERPF) are measured by constant infusion of radio labeled tracers, ^{125}I-iothalamate, and ^{131}Ihippurate, respectively, the patients being in a quiet room, in the semi-supine position. After drawing a blank blood sample, a priming solution containing 0.04 mL/kg body weight of the infusion solution (0.04 MBq of ^{125}Iiothalamate and 0.03MBqof ^{131}I-hippurate) plus an extra of 0.06 MBq of ^{125}I-iothalamate is given at 0800

hours, followed by infusion at 12 mL/h. In order to attain stable plasma concentrations of both tracers, a 2-hour stabilization period followed, after which baseline measurements start at 1000 hours. The clearances are calculated as (U×V)/P and (I×V)/P, respectively. U×V represents the urinary excretion of the tracer, I×V represents the infusion rate of the tracer, and P represents the tracer value in plasma at the end of each clearance period. This method corrects for incomplete bladder emptying and dead space, by multiplying the urinary clearance of ^{125}I-iothalamate with the ratio of the plasma and urinary clearance of ^{131}I-hippurate. The filtration fraction (FF) is calculated as the ratio of GFR and ERPF and expressed as percentage. This measurement is the Gold Standard for renal hemodynamics and routinely performed in the University Medical Center of Groningen (UMCG).

Blood pressure measurements

At start and end of each treatment period, blood pressure is measured over a period of 24hr by an ambulant device (Spacelab). Furthermore, blood pressure is measured at 1-minute intervals for 15 minutes with a semi-automatic device (Dinamap; DE Medical systems, Milwaukee, WI) at study inclusion and during renal hemodynamic measurements. In addition, blood pressure is measured 2 weeks after start of each treatment period and 3 weeks after start of each wash-out period.

24hr urine collection

Patients are asked to collect 24hr urine at study and at start and end of each treatment period to monitor microalbuminuria and measure biomarkers. In total, 24hr urine is collected 5 times in course of this study.

Venapuncture

Blood samples are obtained by venapuncture at study inclusion (20 cc) and at start and end of each treatment period (each 50 cc). Furthermore, 2 weeks after start of each treatment period, a venapuncture is performed to check serum potassium (4 cc). At the end of this study, approximately 230 cc of blood is collected from each patient.

Laboratory measurements

All routine laboratory measurements of this study will be assessed at the laboratory of the University Medical Center Groningen (UMCG).

7.4 Withdrawal of individual subjects

Study drug must be discontinued for a given patient if the investigator determines that continuing would result in a significant safety risk for that patient or if he/she thinks that continuation would be detrimental to the patient’s well-being.

7.4.1 Specific criteria for action and withdrawal

Uncontrolled hypertension According to criteria written in study design (paragraph 3) systolic and diastolic blood pressure must remain lower than 180 and 110 mmHg, respectively. When blood pressure exceeds the before mentioned values during run-in, the patient will be considered a screen failure and prior antihypertensive medication is restarted. If the blood pressure exceeds these values during the study despite rescue medication, the patient will be withdrawn from the study (criteria adapted from ARIA-study: METc 2010.114).

Hypotension Two weeks after start of treatment period, blood pressure is measured for 15 minutes using a semi-automatic device (Dinamap). Hypotension is defined as systolic blood pressure <100 mmHg or a diastolic blood pressure <60 mmHg. Patients with hypotension and orthostatic complaints will be closely monitored and evaluated during the course of the study. If clinically warranted, these patients should be permanently discontinued from the study (criteria adapted from ARIA-study: METc 2010.114).

Hyperkalemia Two weeks after start of treatment period, a venapuncture is performed to check for serum potassium level. A potassium levels above 5.5 mmol/L needs to be reassessed with a second venapuncture within 5 days. After reassessment, patients with a serum potassium level between 5.0 and 6.0 mmol/L will start with dieatary potassium restriction and serum potassium is checked every 2-weeks. A serum potassium ≥6.0 mmol/L will in all cases result in immediate discontinuation of study medication (criteria adapted from ARIA-study: METc 2010.114).

All patients who discontinue study drug, including those who refuse to return for a final visit, will be contacted for safety evaluations during the 30 days following the last dose of study drug. Patients who discontinue study drug should be considered withdrawn from the study after the final visit assessments are performed or when it is clear that the patient will not return for these assessments. Patients who discontinue during the treatment period should be scheduled for a visit as soon as possible, at which time all of the assessments listed for the final visit will be performed.

7.5 Replacement of individual subjects after withdrawal

Patients that meet inclusion criteria will be invited for participation in the study. When patient agrees to participate in the study, informed consent is signed and the patient will enrol in the wash-out period. The patient is included in the study when blood pressure values are within the predefined window (see paragraph 3). When patients decide to

withdraw after start of the treatment period, no new patients can be admitted to the study due to a limited availability of study medication. Although, in our sample size calculation we have taken into account a certain amount of withdrawal and according to our experience, we don't expect withdrawal to exceed this number.

7.6 Follow-up of subjects withdrawn from treatment

Subjects who withdraw from the study will be followed up according to the routine terms of patient care at the outpatient clinic or referred back to their general practitioner.

7.7 Premature termination of the study

There are no predefined criteria for premature termination of the study. If, however, during the conductance of the study information becomes available showing that continuation of the study would result in a significant safety risk for the patients, the principal investigator and project leader will decide to terminate the study.

8 SAFETY REPORTING

8.1 Section 10 WMO event

In accordance to section 10, subsection 1, of the WMO, the investigator will inform the subjects and the reviewing accredited METC if anything occurs, on the basis of which it appears that the disadvantages of participation may be significantly greater than was foreseen in the research proposal. The study will be suspended pending further review by the accredited METC, except insofar as suspension would jeopardise the subjects' health. The investigator will take care that all subjects are kept informed.

8.2 Adverse and serious adverse events

Adverse events are defined as any undesirable experience occurring to a subject during a clinical trial, whether or not considered related to the investigational drug. All adverse events reported spontaneously by the subject or observed by the investigator or his staff will be recorded.

A serious adverse event is any untoward medical occurrence or effect that at any dose results in death;

- is life threatening (at the time of the event);
- requires hospitalisation or prolongation of existing inpatients' hospitalisation;
- results in persistent or significant disability or incapacity;
- is a congenital anomaly or birth defect;
- is a new event of the trial likely to affect the safety of the subjects, such as an unexpected outcome of an adverse reaction, lack of efficacy of an IMP used for the treatment of a life threatening disease, major safety finding from a newly completed animal study, etc.

All SAEs will be reported to the accredited METC that approved the protocol, according to the requirements of that METC.

8.2.1 Suspected unexpected serious adverse reactions (SUSAR)

Adverse reactions are all untoward and unintended responses to an investigational product related to any dose administered.

Unexpected adverse reactions are adverse reactions, of which the nature, or severity, is not consistent with the applicable product information (e.g. Investigator's

Brochure for an unapproved IMP or Summary of Product Characteristics (SPC) for an authorised medicinal product).

The sponsor will report expedited the following SUSARs to the METC:

– SUSARs that have arisen in the clinical trial that was assessed by the METC;
– SUSARs that have arisen in other clinical trial of the same sponsor and with the same medicinal product, and that could have consequences for the safety of the subjects involved in the clinical trial that was assessed by the METC.

The remaining SUSARs are recorded in an overview list (line-listing) that will be submitted once every half year to the METC. This line-listing provides an overview of all SUSARs from the study medicine, accompanied by a brief report highlighting the main points of concern.

The sponsor will report expedited all SUSARs to the competent authority, the Medicine Evaluation Board and the competent authorities in other Member States.

The expedited reporting will occur not later than 15 days after the sponsor has first knowledge of the adverse reactions. For fatal or life threatening cases the term will be maximal 7 days for a preliminary report with another 8 days for completion of the report.

There will be three sets of emergency code breaks, one set will be retained by the project leader, one set will be retained by the principal investigator and a set will be forwarded to the pharmacy. The blinded emergency code break contains the details of drug treatment. In an emergency, de code can be unblinded to identify the treatment given to that subject. Unblinding is not to be performed for any reason, other than an emergency where unblinding is required. When the Investigator removes the scratch-off covering he/she must note the date, time and reason for removing it and record this information in the Comments section of the CRF/ on the code-break card (source data). He/she must also immediately inform the project leader and the principal investigator that the code has been broken. Even though the code is broken, any blood samples for safety, pharmacokinetic and pharmacodynamic assessments will continue to be drawn, for at least 24hr following the last dose as long as doing so will not compromise subject welfare.

It is the responsibility of the Investigator to ensure that there is a procedure in place to allow access to the code break cards in case of emergency. Study drug must be discontinued after unblinding but the subject will be followed until resolution of the

adverse event. At the conclusion of the study, the occurrence of any emergency code breaks will be determined after return of all code break reports.

8.2.2 Annual safety report

In addition to the expedited reporting of SUSARs, the sponsor will submit, once a year throughout the clinical trial, a safety report to the accredited METC, competent authority, Medicine Evaluation Board and competent authorities of the concerned Member States.

This safety report consists of:

– a list of all suspected (unexpected or expected) serious adverse reactions, along with an aggregated summary table of all reported serious adverse reactions, ordered by organ system, per study;
– a report concerning the safety of the subjects, consisting of a complete safety analysis and an evaluation of the balance between the efficacy and the harmfulness of the medicine under investigation.

8.3 Follow-up of adverse events

All adverse events will be followed until they have abated, or until a stable situation has been reached. Depending on the event, follow up may require additional tests or medical procedures as indicated, and/or referral to the general physician or a medical specialist.

8.4 Data Safety Monitoring Board (DSMB)

The institution of a DSMB is not suitable to improve the safety of the patients as this is a cross-over study with study duration of only 26 weeks. The absence of a DSMB has been approved in a comparable study of our department (ARIA-study: METc 2010.114). The safety profile of aliskiren has been well characterized and investigated in completed clinical trials that involved 15.000 hypertensive patients. Specific stopping rules for hyperkalemia and hypotension are defined in the protocol to safeguard patients.

STATISTICAL ANALYSIS

8.5 Descriptive statistics

Patients will be described with their demographical and medical data. Further, all outcome variables will be evaluated with descriptive statistical methods. Results will be expressed as median and 95% confidence interval for not-normally distributed parametric data. Normal parametric data will be expressed as mean and standard deviation. A two-sided *P*-value less than 0.05 will be considered statistical significant.

8.6 Univariate analysis

In analyzing the outcome of the study, renal hemodynamics (GFR, ERPF, and FF) and volume status (ECV) are the primary endpoints. Effects of renal hemodynamics will be expressed as percent change from baseline. Effect of aliskiren and ramipril will be analyzed by paired-sample test and linear mixed effect model. Because of the cross-over design of this study, every patient will be their own control.

8.7 Multivariate analysis

We will perform multivariate analysis to take into account the effects of other independent variables on renal hemodynamics and volume status. We will use linear mixed effect model analysis to adjust for these variables.

8.8 Interim analysis

Not applicable

10 ETHICAL CONSIDERATIONS

10.1 Regulation statement

The study will be conducted according to the principles of the Declaration of Helsinki (Seoul, oct 2008) and in accordance with the Medical Research Involving Human Subjects Act (WMO). The Medical Ethical Committee of the University Medical Center in Groningen has to approve the study.

10.2 Recruitment and consent

Patients will be recruited from clinics of specifically selected general practitioners. In addition, patients will be recruited from the outpatient clinic of the department of internal medicine of the University Medical Center of Groningen (UMCG). Patients will be invited to participate in the study by sending a letter. In this letter, patients will find a full explanation of the study, advantages and disadvantages of participating, and contact information of the research team members working on this study. Moreover, the letter contains contact information of an independent physician, to whom subjects can address questions about the research before, during and after a study. The patients will be given 2 weeks to sign and send back their written informed consent.

10.3 Objection by minors or incapacitated subjects

No minors or incapacitated adults will be included in this study.

10.4 Benefits and risks assessment, group relatedness

There are no direct benefits for the patients to be included. Participation in the study is on a free-will base. Patients will receive restitution of all costs of transportation and a financial support of 200 euro as compensation. Patients will not receive priority for treatment of other diseases in the clinic during this study. Participation in the proposed study is accompanied with only minor risks, if any at all. The blood samples will be drawn by means of venapuncture that will be performed during the visit to the outpatient clinic. Renal hemodynamic measurements using radioactive isotopes are routinely performed in this hospital. The dosage of isotopes used for these renal function measurements are comparable to a plain X-ray (ref 24) and a recent published review showed no evidence of a possible toxic effect of these isotopes (ref 25). All further performed measurements are non-invasive and therefore only minor risks are associated with participation.

10.5 Compensation for injury

The investigator has a liability insurance which is in accordance with article 7, subsection 6 of the WMO.

The University Medical Center Groningen (UMCG) has an insurance which is in accordance with the legal requirements in the Netherlands (Article 7 WMO and the Measure regarding Compulsory Insurance for Clinical Research in Humans of 23th June 2003). This insurance provides cover for damage to research subjects through injury or death caused by the study. The insurance applies to the damage that becomes apparent during the study or within 4 years after the end of the study. All patients will receive written information about this insurance.

10.6 Incentives

Participation of patients in the study is a free-will decision. Patients will receive restitution of all costs for transportation and a financial support of 200 euro. Patients do not receive priority for treatment of other diseases in the clinic during this clinical trial.

11 *A*DMINISTRATIVE ASPECTS AND PUBLICATION

11.1 Handling and storage of data and documents

A subject identification code list will be made to link the data to the subject in order to be able to trace data to an individual subject. This code will not be based on the patient initials and birth-date. The key to the code will be safeguarded by the investigator since the data will be kept for a maximum period of 15 years. The handling of personal data will comply with the Dutch Personal Data Protection Act (in Dutch: De Wet Bescherming Persoonsgegevens, Wbp).

11.2 Amendment

A 'substantial amendment' is defined as an amendment to the terms of the METC application, or to the protocol or any other supporting documentation, that is likely to affect to a significant degree:

- the safety or physical or mental integrity of the subjects of the trial;
- the scientific value of the trial;
- the conduct or management of the trial; or
- the quality or safety of any intervention used in the trial.

All substantial amendments will be notified to the METC and to the competent authority.

Non-substantial amendments will not be notified to the accredited METC and the competent authority, but will be recorded and filed by the sponsor.

11.3 Annual progress report

The sponsor/investigator will submit a summary of the progress of the trial to the accredited METC once a year. Information will be provided on the date of inclusion of the first subject, numbers of subjects included and numbers of subjects that have completed the trial, serious adverse events/ serious adverse reactions, other problems, and amendments.

11.4 End of study report

The sponsor will notify the accredited METC and the competent authority of the end of the study within a period of 90 days. The end of the study is defined as the last patient's last visit. In case the study is ended prematurely, the sponsor will notify the accredited METC and the competent authority within 15 days, including the reasons for the premature termination.

Within one year after the end of the study, the investigator/sponsor will submit a final study report with the results of the study, including any publications/abstracts of the study, to the accredited METC and the Competent Authority.

11.5 Public disclosure and publication policy

Publication policy is in agreement with the CCMO publication statement. Nor the sponsors, nor the principal investigator has a right of veto regarding the way of publishing the results.

References

(1) Bosma RJ, Krikken JA, Homan van der Heide JJ, De Jong PE, Navis GJ. Obesity and renal hemodynamics. Contrib Nephrol 2006;151:184-202.

(2) Kramer AB, Laverman GD, van GH, Navis G. Inter-individual differences in anti-proteinuric response to ACEi in established adriamycin nephrotic rats are predicted by pretreatment renal damage. J Pathol 2003 Sep;201(1):160-7.

(3) Alexander JW, Goodman HR, Hawver LR, Cardi MA. Improvement and stabilization of chronic kidney disease after gastric bypass. Surg Obes Relat Dis 2009 Mar;5(2):237-41.

(4) Soto FC, Higa-Sansone G, Copley JB, Berho M, Kennedy C, LoMenzo E, et al. Renal failure, glomerulonephritis and morbid obesity: improvement after rapid weight loss following laparoscopic gastric bypass. Obes Surg 2005 Jan;15(1):137-40.

(5) Anderson S, Diamond JR, Karnovsky MJ, Brenner BM. Mechanisms underlying transition from acute glomerular injury to late glomerular sclerosis in a rat model of nephrotic syndrome. J Clin Invest 1988 Nov;82(5):1757-68.

(6) Heeg JE, De Zeeuw D, de Jong P. The effects of lisinopril on renal hemodynamics in patients with renal disease. Current opinion in Cardiology 1989.

(7) Laverman GD, Remuzzi G, Ruggenenti P. ACE inhibition versus angiotensin receptor blockade: which is better for renal and cardiovascular protection? J Am Soc Nephrol 2004 Jan;15 Suppl 1:S64-S70.

(8) Apperloo AJ, de Zeeuw D, de Jong PE. A short-term antihypertensive treatment-induced fall in glomerular filtration rate predicts long-term stability of renal function. Kidney Int 1997 Mar;51(3): 703-7.

(9) van Paassen P, De Zeeuw D, De Jong PE. Renal and systemic effects of the renin inhibitor remikiren in patients with essential hypertension. J Cardiovasc Pharmacol 1995 Jul;26(1):39-45.

(10) Apperloo AJ, de Zeeuw D, de Jong PE. Discordant effects of enalapril and lisinopril on systemic and renal hemodynamicss. Clin Pharmacol Ther 1994 Dec;56:647-58.

(11) Fisher NDL, Danser J, Nussberger J, Dole WP, Norman K. Renal and hormonal responses to direct renin inhibition with aliskiren in healthy humans. Circulation 200; 117:3199-205.
