# Supplementary material for: Effects of Direct Renin Blockade on Renal & Systemic Hemodynamics and on RAAS Activity, in Weight Excess and Hypertension: A Randomized Clinical Trial
Source: PLoS One. 2017 Jan 24;12(1):e0169258. doi: 10.1371/journal.pone.0169258 (PMC5261569; doi:10.1371/journal.pone.0169258)
Supplement: S1 Table — (DOCX) [file pone.0169258.s003.docx]

|  | **All subjects**  **(n=15)** | **Subjects randomized to start with ACEi**  **(n=7)** | **Subjects randomized to start with ARB**  **(n=8)** |
| --- | --- | --- | --- |
|  |  |  |  |
| Age (years) | 58 (3) | 58 (6) | 58 (6) |
| Male gender, n (%) | 15 (100%) | 7 (100%) | 8 (100%) |
| BMI (Kg/m^2^) | 30 (1) | 30 (1) | 30 (1) |
| Obesity, n (%) | 7 (47%) | 3 (43%) | 4 (50%) |
| Office SBP (mmHg) | 149 (5) | 157 (8) | 143 (4) |
| Office DBP (mmHg) | 93 (3) | 98 (4) | 88 (3) |
| AHM prior to inclusion, n [range] | 1 [0-2] | 1 [0-2] | 1 [0-2] |
| Waist circumference (cm) | 108 (2) | 108 (3) | 107 (2) |
| Hip circumference (cm) | 103 (2) | 101 (3) | 106 (2) |
| WHR | 1.06 (0.02) | 1.03 (0.02) | 1.07 (0.04) |
| HbA1C (%) | 5.8 (0.2) | 5.7 (0.2) | 6.1 (0.2) |
| Fasting plasma glucose (mmol/L) | 5.9 (0.3) | 5.6 (0.3) | 6.2 (0.4) |
| Total cholesterol (mmol/L) | 4.9 (0.2) | 4.7 (0.3) | 5.1 (0.3) |
| LDL cholesterol (mmol/L) | 3.2 (0.2) | 3.1 (0.2) | 3.4 (0.3) |
| HDL cholesterol (mmol/L) | 1.2 (0.1) | 1.1 (0.1) | 1.3 (0.1) |
